# Sentinel Lymph Node Sampling in Early‐Stage Cervical Cancer: A Cross‐Sectional Survey

**DOI:** 10.1111/1471-0528.70268

**Published:** 2026-05-22

**Authors:** Aakriti Aggarwal, Philip Rolland, Elaine Leung, Jo Morrison, John Butler, Sean Kehoe, Hooman Soleymani majd

**Affiliations:** ^1^ Department of Gynaecology Milton Keynes University Hospital Milton Keynes UK; ^2^ Department of Gynaecology Oncology Gloucestershire Hospitals NHS Foundation Trust Gloucester UK; ^3^ Institute of Cancer and Genomic Sciences, College of Medical and Dental Sciences University of Birmingham Birmingham UK; ^4^ Department of Gynaecology Oncology Somerset NHS Foundation Trust Taunton UK; ^5^ Department of Gynaecology Oncology The Royal Marsden Hospital London UK; ^6^ Department of Gynaecology Oncology Oxford University Hospitals NHS Foundation Trust Oxford UK

Sentinel lymph node (SLN) sampling is increasingly used to reduce surgical morbidity while preserving oncological safety in selected patients with early‐stage cervical cancer. Evidence from prospective studies and international guidance supports SLN sampling in FIGO stage IA1 disease with lymphovascular space invasion and stage IA2–IB1 tumours < 2 cm [1, 2, 3]. Recent BJOG publications have emphasised the importance of surgical de‐escalation and careful implementation of evolving evidence in the management of early‐stage cervical cancer, reinforcing interest in techniques such as SLN sampling. However, the extent to which this approach has been integrated into routine practice in the UK is unclear. We conducted a national survey to explore current use of SLN sampling in early‐stage cervical cancer and to identify perceived barriers to its implementation.

A cross‐sectional, anonymised online survey was developed by UK gynaecological oncologists and reviewed by the British Gynaecological Cancer Society (BGCS) survey committee. The questionnaire included domains on SLN utilisation, indications, techniques, and perceived barriers, using multiple‐choice and free‐text items with conditional branching logic (see Appendix S1 for the full survey instrument). The survey was distributed by BGCS via email to all members with registered active email addresses in two rounds over 6 weeks (May–June 2024). The survey was distributed to 460 BGCS members with registered active email addresses, including gynaecological oncologists, clinical nurse specialists, and clinicians with a specialist interest in gynaecological oncology. Thirty‐two responses were received (overall response rate 7.0%); the proportion of eligible gynaecological oncologists among invitees could not be determined. As survey distribution was managed centrally, individual eligibility screening was not possible. Responses were analysed descriptively, with denominators reported per item. Formal ethics approval was not required, as this was an anonymised survey of healthcare professionals with no patient‐level data collected, in accordance with UK guidance [4].

Thirty‐two responses were received from across the UK, predominantly from consultant gynaecological oncologists. Twenty‐one respondents (66%) reported that SLN sampling was used in their centre for at least one gynaecological malignancy. However, only 14 of these (67%) reported using SLN sampling for early‐stage cervical cancer. Among respondents not routinely using SLN sampling for cervical cancer, the most frequently reported barriers were perceived insufficient evidence, lack of clear national guidance or protocols, limited access to pathological ultra‐staging, and restriction of SLN sampling to research or trial settings (Table 1). Indocyanine green was the most commonly used mapping agent among SLN users. All respondents who did not currently use SLN sampling indicated willingness to adopt the technique in the future, and all respondents expressed willingness to contribute to a prospective audit or national database evaluating surgical management of early‐stage cervical cancer.

**TABLE 1:** Use of sentinel lymph node (SLN) sampling and perceived barriers among respondents.

Characteristic	*n*/*N* (%)
Total respondents	32
Centres using SLN sampling for any gynaecological malignancy	21/32 (66%)
Centres using SLN sampling for early‐stage cervical cancer	14/21 (67%)
Respondents not using SLN sampling for cervical cancer	18/32 (56%)
**Reported barriers to SLN sampling** *	
Perceived insufficient evidence	12/18 (67%)
Lack of clear national guidance/protocols	10/18 (56%)
Limited access to pathological ultra‐staging	9/18 (50%)
Restriction to trial/research settings	8/18 (44%)
Willingness to adopt SLN sampling in future	18/18 (100%)
Willingness to contribute to prospective audit/database	32/32 (100%)

* Multiple responses permitted.

This exploratory survey suggests that, among respondents, SLN sampling is well established for other gynaecological malignancies but remains inconsistently adopted for early‐stage cervical cancer. Interpretation is limited by the small sample size, low response rate, and potential non‐response bias, and the findings should not be considered representative of national practice. Nevertheless, the results highlight structural and evidentiary barriers that may be amenable to intervention. Clearer national guidance, improved access to ultra‐staging, targeted training opportunities, and prospective data collection may support more consistent implementation of SLN sampling in appropriately selected patients. These considerations are likely to be relevant to other healthcare systems integrating evolving surgical evidence into routine clinical practice [4, 5].

## Author Contributions

A.A. and H.S.m. conceived the study and developed the survey questionnaire. E.L., P.R., J.B., J.M., and S.K. contributed to questionnaire design and refinement. A.A. performed data analysis. A.A. and H.S.m. drafted the manuscript. All authors critically reviewed the manuscript for important intellectual content, approved the final version, and agree to be accountable for all aspects of the work.

## Funding

The authors have nothing to report.

## Ethics Statement

The authors have nothing to report.

## Conflicts of Interest

S.K. is a trustee of OVACOME charity; J.M. is co‐chair of BGCS guidelines subgroup.

## Supporting information


**Appendix S1:** bjo70268‐sup‐0001‐AppendixS1.pdf.

## Data Availability

The data that support the findings of this study are available from the corresponding author upon reasonable request.
