# Supplementary material for: Sentinel Lymph Node Sampling in Early‐Stage Cervical Cancer: A Cross‐Sectional Survey
Source: BJOG. 2026 May 22;133(12):2118–9. doi: 10.1111/1471-0528.70268 (PMC13659380; doi:10.1111/1471-0528.70268)
Supplement: Supplementary file 1 [file BJO-133-2118-s001.pdf]

Appendix S1

BGCS Early Stage Cervical Cancer Management Survey for Gynaecology Oncologists (2024)

Thank you for taking the time to fill out this survey. In light of the recent trials published, we would like to collect the trends in practice for surgical management of early-stage cervical cancer in the UK and sentinel LN sampling utilisation.
Note- Multiple options can be selected in some questions. This survey is anonymous.
(Please see reference below for hysterectomy classification)

* Indicates required question

Classification of hysterectomy (for Ca Cervix)

Table : Classification of hysterectomy for invasive cervix cancer according to Querleu and Morrow in 2008

**Type A**<br>Extrafascial hysterectomy - Minimum resection of paracervix (corresponds to the type I RH)	Lateral parametrium removed to the ureter<br>Ureter not tunneled<br>Anterior and posterior parametrium not removed<br>Vessels removed maximally close to the uterus<br>Vaginal resection is minimal without removal of the paracolpos
**Type B**<br>Transection of paracervix at the ureter (corresponds to the type II RH)	Ureter tunneled<br>Partial resection of utero-sacral and vesico-uterine ligaments<br>Resection of para-cervical ligament at the level of ureteral tunnel
**Type C**<br>Transection of paracervix at junction with internal iliac vascular system (corresponds to type III RH)	Transection of lateral parametrium to the iliac vessels<br>Transection of vesico-uterine ligament at the bladder<br>Transection of utero-sacral ligament at the rectum<br>Ureter completely mobilized
**Type C1**<br>**With autonomic nerve sparing/ preservation**	Lateral parametrium – preservation of splanchnic nerves<br>Ventral parametrium – preservation of vesical branch of pelvis plexus<br>Dorsal parametrium – preservation of hypogastric nerve
**Type C2**<br>**Without autonomic nerve sparing/ preservation**	Lateral parametrium - intersection of the splanchnic nerve<br>Ventral parametrium – intersection of vesical branch of pelvic plexus<br>Dorsal parametrium – all branches of hypogastric nerve are dissected
**Type D**<br>**Lateral parametrectomy:**	The line of resection runs between internal obturator internus muscle and lumbosacral plexus

Source: adapetd of Querleu D, Morrow CP. Classification of radical hysterectomy. Lancet Oncol 2008;9(3):297-303.

1. Please select your job profile *

Mark only one oval.

- ⬭ Consultant Gynaecology oncologist with less than 5 years experience *Skip to question 2*
- ⬭ Consultant Gynaecology oncologist with experience ranging between 5 to 10 years *Skip to question 2*
- ⬭ Consultant Gynaecology oncologist with experience of more than 10 years *Skip to question 2*
- ⬭ I am not a Consultant Gynaecology oncologist *Skip to question 2*

Unfortunately you do not fulfil the eligibility criteria for this survey.

We thank you for your interest and time. Please click Submit.

Practice region

2. Which region of the UK do you practice in? *

Mark only one oval.

- ⬭ Northern Ireland
- ⬭ Scotland
- ⬭ Wales
- ⬭ England

Non Fertility sparing surgery

3. For patients diagnosed with **Stage 1a1 Ca Cervix, LVSI negative not requiring fertility preservation** what primary procedure do you usually perform? (Multiple options can be selected)

Tick all that apply.

- [ ] Abdominal approach simple hysterectomy
- [ ] Laparoscopic approach simple hysterectomy
- [ ] Robot assisted simple hysterectomy
- [ ] LLETZ / Conization
- [ ] Other: ______________________

4. For patients diagnosed with **Stage 1a1 Ca Cervix , LVSI Positive ,not requiring fertility preservation** what primary procedure do you usually perform? (Multiple options can be selected)

Tick all that apply.

- [ ] Abdominal approach simple hysterectomy
- [ ] Laparoscopic approach simple hysterectomy
- [ ] Robot assisted simple hysterectomy
- [ ] LLETZ / Conization
- [ ] Other: ______________________

5. For patients diagnosed with **Stage 1a2 /1b1 Ca Cervix, LVSI Negative,** what Lymph node assessment procedure do you usually perform?

Tick all that apply.

	Open Approach	Laparoscopic approach
**Sentinel lymph node (SLN) sampling**	☐	☐
**Bilateral pelvic lymph node dissection (BPLND)**	☐	☐
**SLN sampling + BPLND**	☐	☐

6. For patients diagnosed with **Stage 1a2 Ca Cervix, LVSI negative, not requiring fertility preservation** what primary procedure do you usually perform? (Multiple options can be selected)

Tick all that apply.

	Open Approach	Laparoscopic Approach	Robotic assisted approach
**LLETZ/ Conization (Please ignore approach/ select any option)**	☐	☐	☐
**Type A/ Simple total hysterectomy**	☐	☐	☐
**Type B/ Semiradical hysterectomy**	☐	☐	☐
**Type C1/ Nerve sparing radical hysterectomy**	☐	☐	☐
**Type C2/ Radical hysterectomy**	☐	☐	☐

7. For patients diagnosed with **Stage 1a2 Ca Cervix, LVSI positive, not requiring fertility preservation** what primary procedure do you usually perform? (Multiple options can be selected)

Tick all that apply.

	Open Approach	Laparoscopic Approach	Robotic assisted approach
**Type A/ Simple total hysterectomy**	☐	☐	☐
**Type B/ Semiradical hysterectomy**	☐	☐	☐
**Type C1/ Nerve sparing radical hysterectomy**	☐	☐	☐
**Type C2/ Radical hysterectomy**	☐	☐	☐

8. For patients diagnosed with **Stage 1b1 Ca Cervix, Lymph node negative (on imaging), not requiring fertility preservation** what primary procedure do you usually perform? (Multiple options can be selected)

Tick all that apply.

	Open Approach	Laparoscopic Approach	Robotic assisted approach
**Type A/ Simple total hysterectomy**	☐	☐	☐
**Type B/ Semiradical hysterectomy**	☐	☐	☐
**Type C1/ Nerve sparing radical hysterectomy**	☐	☐	☐
**Type C2/ Radical hysterectomy**	☐	☐	☐

9. Would you be happy to answer a few more questions regarding fertility sparing surgery and sentinel LN sampling for early stage cervical cancer? *

Mark only one oval.

- ◯ Yes *Skip to question 10*
- ◯ No

Fertility sparing surgery + SLN sampling survey

10. For a patient diagnosed with **Stage 1a1 Ca Cervix, LVSI negative, requesting fertility preservation**, what procedure do you routinely perform? (Multiple options can be selected)

Tick all that apply.

- [ ] LLETZ
- [ ] Conization
- [ ] Other: ______________________

11. For patients diagnosed with **Stage 1a1 Ca Cervix, LVSI Positive, requiring fertility preservation** what primary procedure do you routinely perform? (Multiple options can be selected)

Tick all that apply.

- [ ] LLETZ + laparoscopic BPLND
- [ ] LLETZ + open adominal approach BPLND
- [ ] LLETZ + laparoscopic SLN sampling (+/- BPLND)
- [ ] LLETZ + open abdominal approach SLN sampling (+/- BPLND)
- [ ] Other: ______________________

12. For patients diagnosed with **Stage 1a2 Ca Cervix , LVSI Negative, requiring fertility preservation** what procedure do you usually perform? (Multiple options can be selected)

Tick all that apply.

- [ ] LLETZ/ conization/ simple trachelectomy
- [ ] LLETZ/ conization/ simple trachelectomy + SLN sampling (laparoscopic)
- [ ] LLETZ/ conization/ simple trachelectomy+ SLN sampling (open approach)
- [ ] LLETZ/ conization/ simple trachelectomy + BPLND (laparoscopic)
- [ ] LLETZ/ conization/ simple trachelectomy+ BPLND (open approach)
- [ ] Other: ______________________

13. NoFor patients diagnosed with **Stage 1a2 Ca Cervix, LVSI POSITIVE, requiring fertility preservation** what primary procedure do you usually perform (excluding Lymph node assessment)? (Multiple options can be selected)

Mark only one oval.

- LLETZ / Conization
- Simple trachelectomy + cerclage (laparoscopic approach)
- Simple trachelectomy +cerclage (open approach)
- Other: ______________________

14. For patients diagnosed with **Stage 1b1 Ca Cervix, LN negative (on imaging), requiring fertility preservation** what primary procedure do you usually perform? (Multiple options can be selected)

Tick all that apply.

	Open approach	Laparoscopic approach	Robot assisted approach
**LLETZ/Conization (please ignore approach, select any option)**	☐	☐	☐
**Simple trachelectomy + cerclage**	☐	☐	☐
**Radical trachelectomy + cerclage**	☐	☐	☐

15. Does your centre perform/offer sentinel LN sampling?

Mark only one oval.

- Yes *Skip to question 18*
- No *Skip to question 16*

Barriers to SLN sampling

16. In your opinon, what are the barriers to performing Sentinel lymph node sampling for gynaecology cancer in your institution? (Multiple options can be selected)

Tick all that apply.

- [ ] Insufficient evidence to support
- [ ] Lack of guidance / protocol
- [ ] It should only be done as part of a trial and there is no current trial
- [ ] No access to ultra staging
- [ ] Unfamiliar with the technique
- [ ] Other: ______________________

17. If your centre does not currently offer sentinel LN sampling- Would you be keen to transition from BPLND to SLN sampling if this option is available in the future?

Mark only one oval.

- ( ) Yes
- ( ) No

SLN sampling

18. Which SLN technique does your institute routinely perform (can select multiple options)

Tick all that apply.

- [ ] ICG (Indocyanine green)
- [ ] Tecnitium 99m (Tc99m)
- [ ] ICG as part of trial
- [ ] Tc 99m as part of trial
- [ ] Methylene blue dye
- [ ] Other: ______________________

19. For which gynaecological cancers do you routinely perform Sentinel lymph node sampling? (Multiple options can be selected)

Tick all that apply.

- [ ] Low grade endometrial cancer (MRI predicted 1a)
- [ ] Low grade endometrial cancer (MRI predicted 1b/2)
- [ ] All low grade endometrial cancer
- [ ] High grade endometrial cancer
- [ ] Vulval cancers <4cm
- [ ] Ovarian cancers
- [ ] Cervical cancers 1b1/2

20. In your opinion, what are the barriers to performing Sentinel lymph node sampling for CERTAIN gynaecology cancer in your institution? (Multiple options can be selected)

Mark only one oval.

- ◯ Insufficient evidence to support
- ◯ Lack of guidance / protocol
- ◯ It should only be done as part of a trial and there is no current trial
- ◯ No access to ultra staging / lack of pathology support
- ◯ Unfamiliar with the technique
- ◯ NOT APPLICABLE

21. Would you be willing to participate in a National [BGCS] prospective audit/database for early-stage cervical cancer management

Mark only one oval.

- ◯ Yes
- ◯ No

This content is neither created nor endorsed by Google.
